# Approaches for Enhancing Wastewater Treatment of Photocatalytic Fuel Cells: A Review

**DOI:** 10.3390/ma17092139

**Published:** 2024-05-02

**Authors:** Penghui Li, Xiaohan Zhou, Haoyi Yang, Yun He, Yujiao Kan, Yang Zhang, Yanan Shang, Yizhen Zhang, Xiaoqiang Cao, Michael K. H. Leung

**Affiliations:** 1 College of Safety and Environmental Engineering, Shandong University of Science and Technology, Qingdao 266590, Chinakanyujiao@sdust.edu.cn (Y.K.); zhangyang@sdust.edu.cn (Y.Z.);; 2 School of Chemical and Environmental Engineering, Wuhan Polytechnic University, Wuhan 430024, China; 3 Institute of Yellow River Delta Earth Surface Processes and Ecological Integrity, Shandong University of Science and Technology, Qingdao 266590, China; 4 Shandong Provincial Key Laboratory of Eco-Environmental Science for Yellow River Delta, Shandong University of Aeronautics, Binzhou 256500, China; 5 Ability R&D Energy Research Centre, School of Energy and Environment, City University of Hong Kong, Hong Kong, China; mkh.leung@cityu.edu.hk

**Keywords:** photocatalytic fuel cell, electrodes, advanced oxidation processes, wastewater treatment

## Abstract

Environmental pollution and energy crises have garnered global attention. The substantial discharge of organic waste into water bodies has led to profound environmental contamination. Photocatalytic fuel cells (PFCs) enabling the simultaneous removal of refractory contaminants and recovery of the chemical energy contained in organic pollutants provides a potential strategy to solve environmental issues and the energy crisis. This review will discuss the fundamentals, working principle, and configuration development of PFCs and photocatalytic microbial fuel cells (PMFCs). We particularly focus on the strategies for improving the wastewater treatment performance of PFCs/PMFCs in terms of coupled advanced oxidation processes, the rational design of high-efficiency electrodes, and the strengthening of the mass transfer process. The significant potential of PFCs/PMFCs in various fields is further discussed in detail. This review is intended to provide some guidance for the better implementation and widespread adoption of PFC wastewater treatment technologies.

## 1. Introduction

Environmental pollution and energy crises represent two formidable challenges confronting the sustainable development of human society [1,2,3,4]. As societal advancement accelerates, the severity of energy and environmental issues continues to escalate. Given the pressing nature of contemporary water pollution challenges, research and development in water treatment technologies assume paramount importance [5,6]. Water treatment processes are typically categorized into physical, chemical, and biological methods based on their operational principles [7,8,9]. Wastewater contains a plethora of organic compounds rich in chemical energy, representing a misallocated resource. However, prevailing conventional water treatment strategies primarily emphasize pollutant removal to achieve regulatory effluent standards [10,11]. The imperative to reduce energy consumption while ensuring efficient wastewater treatment remains an urgent concern [12,13,14].

Fuel-cell-based water pollution control systems represent cutting-edge technologies for wastewater resource utilization, providing simultaneous wastewater treatment and electricity generation [4,15,16,17]. This innovation carries significant implications for tackling water pollution and mitigating energy deficits. Among these technologies, the integration of photocatalytic fuel cells (PFCs) and photocatalytic microbial fuel cells (PMFCs) based on photoelectrochemical and photocatalytic bioelectrochemical processes offers a comprehensive solution characterized by high efficiency, energy conservation, and operational simplicity [18,19,20]. These integrated systems present a promising approach for addressing the treatment of stubborn organic wastewater. He et al. [2] reported the fundamentals and technical advancements of PFCs, with a particular emphasis on novel fuel cell configurations. Meanwhile, the rational design of electrode materials was reviewed, focusing on surface properties, morphology, facet structure, and interface reaction engineering [3]. Ni et al. [21] summarized recent progress in the development of photoanode/photocathode materials, cathodic materials, system configurations, and radical reaction processes, giving five key strategies to enhance the dynamics and charge transfer properties of the constructed system. Additionally, the challenges, perspectives, and future studies were extensively discussed for different PFC systems.

In this review, the fundamentals, working principle, and configuration development of PFCs and photocatalytic microbial fuel cells (PMFCs) were discussed. The strategies for improving the wastewater treatment performance of PFCs/PMFCs in terms of coupled advanced oxidation processes, rational design of high-efficiency electrodes, and strengthening of mass transfer process are highlighted. The significant potential of PFCs/PMFCs in various fields is further discussed in detail. Clearly improving the strategy of the PFC purification of wastewater will help its future application in practical wastewater treatment.

## 2. Fuel Cell System

Traditional wastewater treatment methods, such as adsorption, chemical precipitation, membrane filtration, chemical oxidation, and biodegradation, often require a large amount of energy consumption. In contrast, PFCs could convert chemical energy in wastewater into electrical energy only by using solar energy, while effectively degrading organic pollutants in wastewater without additional energy. In previous studies, German and American research teams summarized and analyzed the power consumption of AOP technology based on EEO indicators [22]. The statistical results showed that the median energy consumption of the electrochemical oxidation method was 38.1 kWh m^−3^, and that of the UV photocatalysis method was 335 kWh m^−3^. In contrast, the use of PFCs/MPFCs to treat pollutants in wastewater effectively reduces energy consumption and enables energy conversion.

### 2.1. PFC

**Figure 1:**
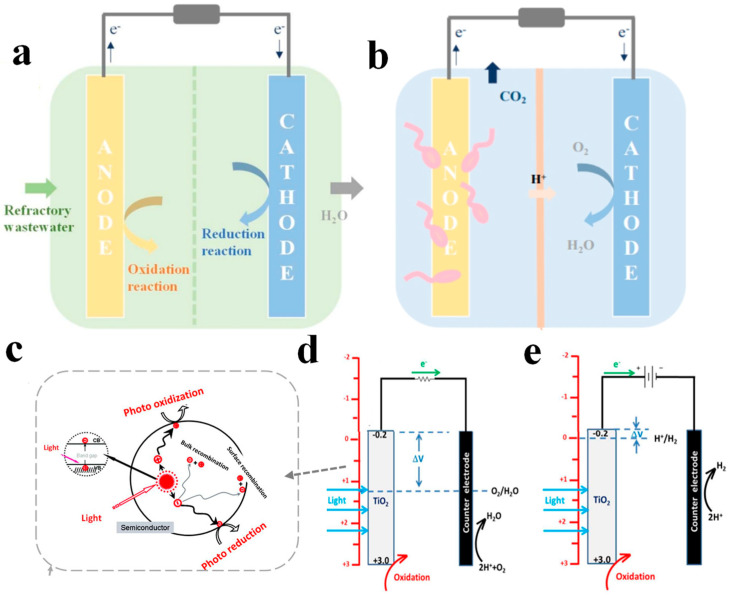
Schematic of (**a**) PFC and (**b**) MFC system, (**c**) photocatalytic reaction process [31], (**d**) PFC system operation without bias, and (**e**) PEC operation with external bias [12].

In 2006, Kaneko et al. [23] introduced the concept of PFCs, integrating photocatalytic technology into a wastewater-fueled fuel cell system, to achieve simultaneous wastewater purification and electricity generation [9,24,25]. The fundamental structure of PFCs comprises a photocatalytic anode, a photo/electrocatalytic cathode, conductive wires connecting to an external resistor, and a reactor filled with reaction solution (Figure 1a). The photocatalytic anode is composed of semiconductor materials and their supporting materials (responsible for conducting electric current), while the cathode consists of electrocatalytic materials loaded with noble metals such as Pt or semiconductor materials with band positions differing from those of the anode [26,27,28]. Through conductive wires, the electrodes connect to the external circuit resistor, forming a closed circuit wherein electrons flow from the anode to the cathode driven by the potential difference [29,30].

### 2.2. PMFCs

The multitude of biological species, characterized by their prolific reproductive capacities and ability to thrive under mild environmental conditions, makes them well suited for sustained, long-term operation. Adequate illumination is a crucial environmental factor for the efficient functioning of PMFCs. In contrast to photo-driven PFC systems, employing microorganisms with electricity-generating properties as anodes allows for the stable operation of fuel cells even in the absence of light. A typical MFC system, as depicted in Figure 1b [31], consists of an anode, a cathode, an external circuit, and separate anode and cathodic chambers partitioned by a proton exchange membrane (PEM).

The integration of microbial technology and photoelectrochemical techniques in PMFCs exploits the synergistic interplay between microbial metabolism and photoelectrocatalysis to convert the chemical energy derived from chemical fuels or complex biomasses into environmentally friendly electrical energy [32,33]. Designing PMFCs requires a comprehensive consideration of the oxidation–reduction potentials of the anodic microorganisms, as well as the electrocatalytic reaction potentials of the cathode or the energy level positions of semiconductor materials. This ensures optimal matching between the biological anode and the photo/electrocatalytic cathode, facilitating the directed transfer of electrons [34,35]. During the metabolic processes of the anodic microorganisms, electrons and protons are simultaneously generated alongside the decomposition of organic compounds. While electrons are transferred through the external circuit to the cathode, protons traverse the PEM to reach the cathodic chamber, where they collectively participate in the cathodic reduction reaction [36,37]. These processes aid in mitigating the recombination of photogenerated holes and electrons during the photocatalytic process, consequently boosting the photocatalytic efficiency of the cathode [38,39]. It enables efficient electricity generation driven by microorganisms and facilitates synergistic photoelectrocatalytic reactions for pollutant degradation. In 2010, researchers reported a PMFC, wherein microorganisms were utilized in an anodic chamber, while hematite-loaded graphite carbon served as the photocatalytic cathode. The study demonstrated that the enhanced electron transfer process at the photocatalytic cathode elevated the power output. Additionally, the photocatalytic activity of the cathode substantially improved the degradation performance of methyl orange. Mohammad et al. [40] further constructed PMFCs by modifying commercial TiO_2_ as photocatalytic cathodes. This modification led to the generation of Ti^3+^ and oxygen vacancies, enhancing its photocatalytic activity and photoelectrochemical behavior under visible light assistance. Consequently, this approach not only improved the electricity generation activity but also enhanced its capability for the catalytic degradation of pollutants.

### 2.3. The Working Principle

As depicted in Figure 1c, semiconductor photocatalytic electrodes undergo a series of events involving the generation, separation, migration, and recombination of photoinduced electrons and holes under solar or artificial light irradiation [41,42]. Upon excitation by incident light with energy exceeding their bandgap width, electrons in the valence band absorb photon energy and transfer to the conduction band, generating photoinduced electrons (e^−^) while leaving photoinduced holes (h^+^) in the valence band. Subsequently, photoinduced electrons and holes migrate to different positions on the material surface, where they participate in oxidation–reduction reactions with adsorbed H_2_O, dissolved oxygen, or pollutants [43,44]. The migration process of photoinduced electrons and holes readily undergoes recombination within the material, accompanied by the release of photon/thermal energy, leading to the deactivation of electrons and holes [45,46]. Surface defects are pivotal in the functionality of photocatalytic materials. Upon illumination, these defects serve as sites for trapping light-excited electrons and holes, facilitating their efficient separation. On the other hand, more reaction sites are provided by surface defects, thereby improving the overall photocatalytic performance [47,48]. Compared to conventional photocatalytic systems, PFCs offer convenient pathways for the transfer of photoinduced electrons. Photoinduced electrons generated at the photoanode traverse through the external circuit to the counter electrode, effectively facilitating the separation of holes and electrons, thus playing a pivotal role in enhancing photocatalytic activity [27,49].

The working principle of PFCs, illustrated in Figure 1d, is exemplified by a typical PFC with an n-type semiconductor TiO_2_ as the photoanode and Pt as the cathode [13]. The photoanode undergoes the aforementioned photocatalytic process, generating electron–hole pairs (Equations (1)–(4)), which migrate from the interior of the electrode material to the surface. Activated electrons react with dissolved oxygen to produce superoxide radicals (•O_2_^−^), while holes react with the water or OH^−^ adsorbed on the photoanode surface to form hydroxyl radicals (•OH). Both radicals and holes exhibit strong oxidation capabilities, enabling the direct degradation of organic pollutants in wastewater, resulting in the generation of small molecules such as CO_2_ and H_2_O [50]. Simultaneously, some electrons transfer from the photoanode surface to the cathode via the external circuit, forming a closed loop [51].

Under aerobic conditions, oxygen in the cathodic chamber is utilized as an electron acceptor to undergo catalytic reduction reactions (Equations (5)–(8)), producing H_2_O_2_ (acidic medium) or HO_2_^−^ (alkaline medium) via the two-electron pathway, and H_2_O (acidic medium) or OH^−^ (alkaline medium) via the four-electron pathway. Under anaerobic conditions, the catalytic cathode reduces protons to undergo hydrogen evolution reactions (Equations (9)–(10)), while bio-cathodes undergo complex bioelectrochemical reactions.

Upon photoexcitation, the directional electron transport is driven by the potential difference formed between the electrodes, maintaining the stable operation of the system and avoiding additional energy consumption. The electrochemical performance of PFCs largely depends on the microstructure and properties of the catalytic electrodes. Therefore, constructing dual electrode materials with matching band structures is crucial for ensuring the stable operation of PFCs.

Anodic reactions in a PFC include the following:

TiO_2_ + hv→h^+^ + e^−^ (1)

h^+^ + H_2_O→•OH + H^+^ (2)

h^+^ + OH^−^→•OH (3)

e^−^ + O_2_→•O_2_^−^ (4)

Under aerobic conditions, the cathodic reaction is as follows:

4H^+^ + 2O_2_ + 4e^−^→2H_2_O (5)

2H^+^ + O_2_ + 2e^−^→H_2_O_2_ (6)

2H_2_O + O_2_ + 4e^−^→4OH^−^ (7)

H_2_O + O_2_ + 2e^−^→HO_2_^−^ + OH^−^ (8)

Under anaerobic conditions, the cathodic reaction is as follows:

2H^+^ + 2e^−^→H_2_ (9)

2H_2_O + 2e^−^→H_2_ + 2OH^−^ (10)

### 2.4. The Configuration

The diverse types of PFCs can be categorized based on their structures into dual-chamber PFCs and single-chamber PFCs; based on their electrode properties, they can be categorized into single-photoelectrode PFCs and dual-photoelectrode PFCs; and they can also be categorized based on their consumed substrate into fuel-consuming PFCs and pollution-controlling PFCs.

**Figure 2:**
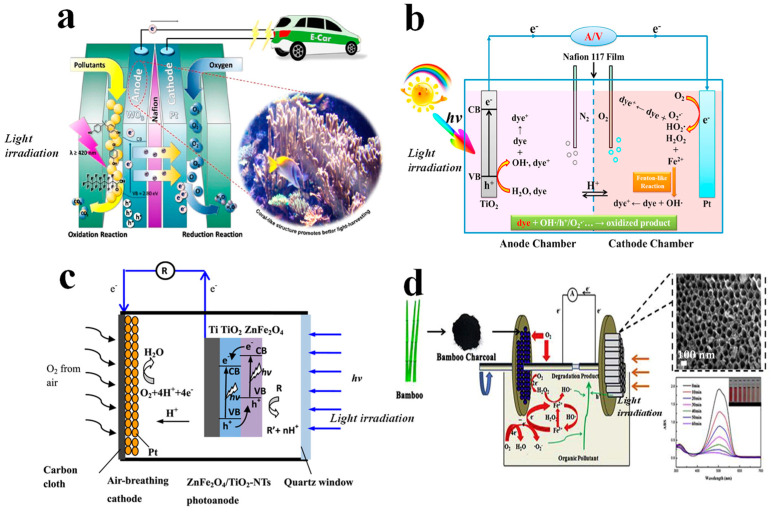
Illustration of (**a**) dual−chamber PFC [52], (**b**) dual−chamber PFC assisted by Fenton−like reactions [60], (**c**) single−chamber PFC with air−breathing cathode [28], and (**d**) single−chamber PFC integrating Fenton process with rotating disk electrodes [55].

The earliest reported dual-chamber PFCs are akin to PEC devices, comprising an anodic chamber, a cathodic chamber, and a PEM, also known as an H-type reactor. The distinguishing feature lies in the separation of the anodic and cathodic chambers by the PEM, ensuring their independent functionalities. Li et al. [52] reported a dual-chamber H-type PFC system utilizing biomimetic porous coral-like WO_3_/W as the photoanode and Pt as the cathode, separated by Nafion membranes (Figure 2a). The unique structural characteristics of the photoanode exhibit the excellent visible-light photocatalytic degradation of persistent organic pollutants, while simultaneous ORR occurs in the cathodic chamber. This system achieved an output power of 0.0013 mW cm^−2^. In comparison to dual-chamber PFCs (Figure 2b) [50], single-chamber PFCs offer simpler configurations and a more convenient operation. Single-chamber PFCs do not require a PEM, effectively reducing system costs, while the reduced electrode distance enhances the mass transfer efficiency. Typical devices include air-cathode single-chamber PFCs, such as air-cathode PFCs with ZnFe_2_O_4_/TiO_2_ composite photoanodes (Figure 2c) [28], WO_3_/FTO photoanodes [53], and CdS/TiO_2_ composite photoanodes [54]. In single-chamber PFCs where both the anode and cathode are placed in the same reaction vessel, catalytic reactions occur in the same solution, facilitating the transport of protons and other species within the system. Additionally, researchers have explored novel PFC configurations such as rotating electrode PFCs [55,56] (Figure 2d), PFCs coupled with electrodialysis technology [57], and microfluidic PFCs [58,59].

PFCs are classified into single-photoelectrode and dual-photoelectrode types based on the cathodic material. In single-photoelectrode PFCs, the photoanode is composed of semiconductor materials, while the cathode comprises electrocatalytic materials. Photocatalytic reactions occur at the photoanode, and the generated photoinduced electrons are transported to the cathode via the external circuit, initiating catalytic reduction reactions on the cathode surface depending on the electron acceptor. Dual-photoelectrode PFCs feature both the anode and cathode made of semiconductor materials with photocatalytic activity. Typically, n-type semiconductors are used for the anode, and p-type semiconductors are employed for the cathode. Under illumination, both the photoanode and photocathode generate photoinduced electrons and holes, and the difference in Fermi energy levels between the electrodes drives directional electron movement. Recent studies successively reported single-photoelectrode PFCs comprising TiO_2_/Ti nanotube array photoanodes and Pt-based cathodes, as well as dual-photoelectrode PFCs composed of a TiO_2_/Ti nanotube array photoanode and Cu_2_O/Cu photocathode [59,61]. Both configurations achieved pollutant degradation and high-performance electricity generation. Notably, single-photoelectrode PFCs exhibited superior electricity generation performance compared to their dual-photoelectrode counterparts [62].

## 3. Strategies for Improving Wastewater Treatment Performance of PFCs

In the case of PFC systems, catalytic reactions encompass light absorption characteristics, the separation and migration of charge carriers, electron transfer between electrodes, anodic oxidation, cathodic reduction reactions, and the mass transfer process. Moreover, in PMFCs, microbial metabolic pathways play a pivotal role. To achieve the high-efficiency treatment of refractory wastewater and energy generation, several strategies can be pursued from a systemic standpoint.

### 3.1. Coupled with Advanced Oxidation Processes

(1) Advanced oxidation processes based on hydroxyl radicals.

**Figure 3:**
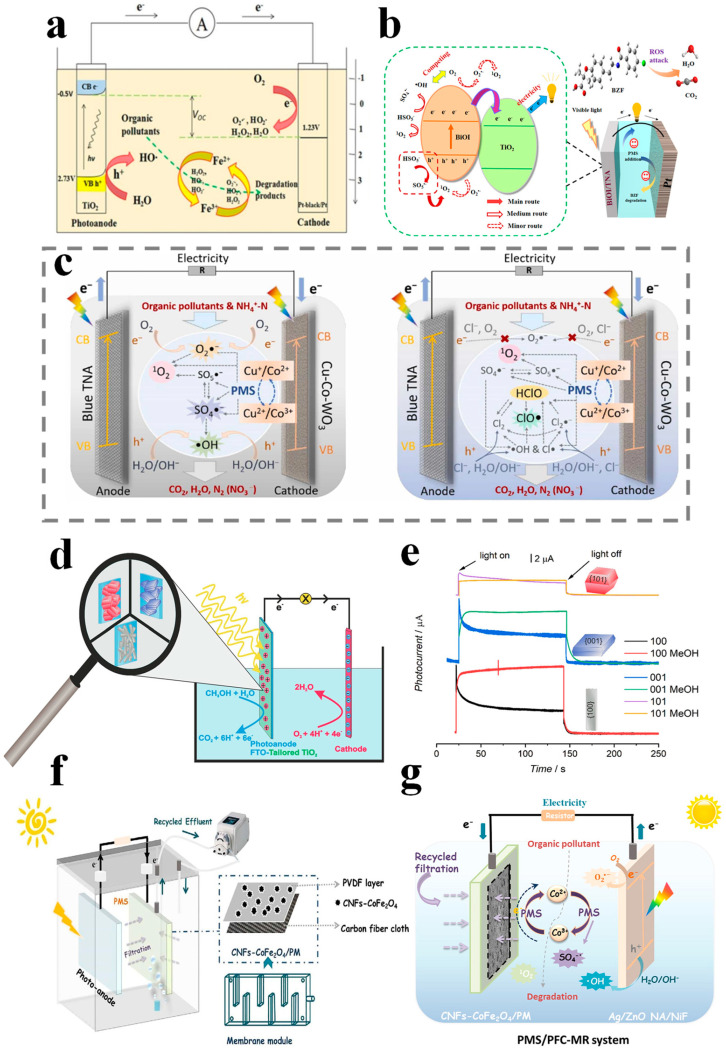
Schematic illustration of (**a**) the mechanism of Fenton−PFC system [67], (**b**) the mechanism of PFC (BiOI/TNA)/PMS/vis system [72], (**c**) the mechanism of PFC/PMS system in fresh−water en−vironment (**left**) and marine environment (**right**), respectively [73], (**d**) the mechanism of PFC with different shape of TiO_2_ crystals as photoanode [74], (**e**) transient photocurrent response of photoanode made of various tailored TiO_2_ crystals, (**f**,**g**) schematic illustration of PMS/PFC−MR [64].

Traditional advanced oxidation processes (AOPs) primarily revolve around the oxidation of diverse pollutants catalyzed by hydroxyl radicals (•OH). The oxidation–reduction potential of •OH can ascend to 2.8 V vs. NHE, showcasing formidable oxidizing prowess capable of swiftly and non-selectively degrading, or even mineralizing, pollutants. Prominent AOPs leveraging •OH encompass Fenton oxidation, photocatalytic oxidation, electrochemical oxidation, ozonation, peroxidation, and ultrasonic oxidation. Recent studies have pioneered the integration of Fenton oxidation technology with PFCs/PMFCs to create innovative wastewater treatment systems. Through the selection of suitable cathode materials to regulate the cathodic two-electron pathway for H_2_O_2_ generation, coupled with the introduction of Fe^2+^ to initiate the Fenton reaction, a Fenton–fuel cell system was established [63]. Leveraging photocatalysis or microbial-driven electricity production can effectively mitigate the energy consumption associated with electro-Fenton techniques. Furthermore, the in situ generation of H_2_O_2_ at the cathode circumvents challenges related to the transportation and storage of H_2_O_2_ encountered in conventional approaches [64]. Feng et al. [65] introduced a Fenton–bioelectrochemical system, where a carbon nanotube/γ-FeOOH composite cathode, driven by microbial anodes, catalyzes the in situ generation of H_2_O_2_ and ferrous ions to initiate the Fenton reaction, leading to the efficient degradation of organic dyes. Subsequently, Fenton-coupled MFC systems have been applied for the degradation of various types of pollutants. Zhou et al. [66,67] has developed different variants of Fenton–PFC coupling systems. In one instance, TiO_2_ was utilized as a photocathode in the Fenton–PFC system. This facilitated catalytic reactions throughout the reaction solution via continuous photoelectro-Fenton processes, thereby enhancing pollutant degradation performance (Figure 3a). Furthermore, the utilization of TiO_2_/WO_3_/W-modified photocathodes in Fenton–PFC systems effectively improved the long-term stability and electricity generation efficiency of the system. Additionally, Xu et al. [57,68,69] enhanced the performance of integrated Fenton–PFC systems by constructing novel Fe-based cathodes (Fe@Fe_2_O_3_/carbon felt, FeVO_4_/carbon felt) and coupling with reverse electrodialysis techniques, eliminating the need for continuous external voltage input and ferrous ion addition. Lee et al. [70] utilized immobilized ZnO/Zn as photoanodes with a Pt/C plate serving as the cathode and investigated the influence of varying initial dye concentrations and pH levels on the decolorization efficiency and power generation of the PFCs. The findings demonstrated that closed-loop PFCs exhibited the enhanced degradation performance of active green 19 compared to open-loop PFCs. Dhawle et al. [16] engineered an integrated reactor capable of catalyzing the generation of H_2_O_2_ under solar light radiation, concurrently with persistent pollutants degradation via a UV/H_2_O_2_ process. The production of H_2_O_2_ occurred through the photocatalytic reduction of atmospheric oxygen within the fuel cells. Despite the promising prospects of Fenton–fuel cell systems, challenges persist regarding power density, H_2_O_2_ concentration, cathode materials, Fe^2+^ concentration, and pH range [71].

(2) Advanced oxidation processes based on sulfate radicals.

Sulfate radicals (SO_4_^•−^) with oxidation–reduction potential (E_0_ = 2.5–3.1 V) indicate favorable oxidative capacity and possess extended radical lifetimes (20–30 μs), enabling a fuller exertion of their oxidizing action. Additionally, sulfate radicals exhibit higher selectivity in oxidation reactions, making them more effective in treating certain types of pollutants [75,76]. These attributes allow SO_4_^•−^ to achieve more extensive contact and reaction with pollutants, thereby enhancing the utilization efficiency and catalytic efficacy of radicals [75,76]. Peroxydisulfates, such as peroxymonosulfate (PMS) and peroxy disulfate (PDS), can generate SO_4_^•−^ through activation processes. Similar to H_2_O_2_, peroxydisulfates contain unstable O-O bonds, necessitating external energy input for O-O bond cleavage and SO_4_^•−^ production.

A collaborative system including PMS activation in a PFC system under visible light (PFC/PMS/vis system) was established, with a BiOI/TiO_2_ nanotube arrays p-n type heterojunction as the photoanode (Figure 3b). The results revealed that adding PMS could promote the generation of radicals. Among them, ^1^O_2_ played a key role in the degradation process, generating from a reaction between PMS and h^+^ [72]. For the advanced removal of marine pollutants, a novel PFC/PMS system was reported (Figure 3c), by using Cu-Co-a WO_3_ catalytic cathode and blue TiO_2_ nanotube arrays (TNAs) anode [73]. Integrated (photo-)electrocatalytic chlorination and PMS–chlorination processes, refractory organic pollutant (removal rate: 94.18%), and inorganic nitrogen (removal rate: 95.73%) were simultaneously removed in natural seawater to a qualified level. Additionally, this PFC/PMS system with an open circuit voltage of 0.57 V provides extra electricity, making this system competitive in energy savings. Nonetheless, compared to heterogeneous PMS catalytic systems utilizing powdered catalysts, additional energy consumption remains a concern. PFC systems possess the capability to generate self-bias between electrodes, facilitating directed electron flow and thereby effectively reducing energy consumption. 

### 3.2. Construction of High-Efficiency Photocatalytic Electrode

By leveraging nanoscale control and exploiting the dimension-related effects of the photocatalytic electrode, its catalytic performance can be optimized. Designing a multi-dimensional photoanode facilitates the expansion of functional diversity and application domains. Consequently, tailoring materials based on dimensions (0D, 1D, 2D) emerges as a crucial strategy for boosting material efficiency and broadening applications. It provides insights into the unique light scattering/capturing behaviors and charge carrier transfer mechanisms exhibited by nanostructured photocatalytic electrodes across varying dimensions [77]. Zero-dimensional photocatalysts (nanoparticles, nanospheres, nanocrystals, quantum dots) are immobilized onto conductive substrates (metallic conductive materials, carbon-based conductive materials, polymer conductive materials), resulting in increased charge carrier recombination probabilities as photo-generated electrons traverse multiple particle interfaces before reaching the conductive substrate. Conversely, 1D nanostructured photocatalytic electrodes involve the direct growth or attachment of well-defined nanostructures onto the surface of conductive substrates, facilitating swift charge carrier transfer and effectively alleviating charge transfer inefficiencies inherent in 0D electrode configurations, thereby reducing light loss. On conductive substrates, 2D nanostructures offer a larger active surface area, thereby enhancing charge carrier separation and transfer at interfaces. The strategic assembly of 0D, 1D, and 2D nanostructured components culminates in the fabrication of 3D nanostructured photocatalytic electrodes, characterized by a structured design that ensures distinctive light-responsive and charge carrier transfer properties [74]. Pan et al. [52] fabricated a photoanode with a bionic porous coral-like nanostructure, significantly enhancing the light-harvesting capacity. This resulted in achieving a maximum photocurrent density of 0.31 mA/cm^2^ and a high incident photon conversion efficiency value of 5.72% under visible light irradiation (λ > 420 nm). Notably, tailored crystals with different shapes and different facets also serve as effective strategies to construct a photoelectrode with ideal photo(electro)catalytic activity. For example, the PFC in Figure 3d was constructed by using a photoanode with anatase crystals of various shapes (tetragonal truncated bipyramids, sheets, and belts), characterized by different major exposed facets ({101}, {001}, and {100}). As a result, photoanodes with belt-shaped crystals dominated by {100} facets exhibit superior photocurrents (Figure 3e).

### 3.3. Strengthen Mass Transfer Capacity

In photocatalytic/microbial fuel cells, both the anode and cathode are pivotal in facilitating photo/biochemical reactions, alongside internal mass transfer processes. These intricate that mass transfer mechanisms exert a direct influence on the rates of photoelectrochemical and electrochemical reactions within the system [78]. Delineation of the fuel cell’s reaction regions is based on distinct photo/electrochemical reaction processes, encompassing the catalytic electrode interface, double charge layer, boundary layer, and electrolyte mainstream region, among others. Within the semiconductor electrode, crucial processes such as light absorption and carrier transport transpire; meanwhile, photoelectrochemical and electrochemical processes unfold at the interface between the photo/electrocatalytic components and the electrolyte solution. Concomitantly, the transport of reactants, products, and ions unfolds in the boundary layer adjacent to the reaction interface and throughout the electrolyte’s mainstream region. Notably, the boundary layer represents an arena characterized by significant alterations in substance concentration and velocity. Here, reactants and ions predominantly access the catalytic electrode surface via convection and diffusion mechanisms; thereafter, reactants become adsorbed onto catalytic particles situated on the electrode surface, prompting catalytic oxidation or reduction reactions. Subsequently, products generated by catalytic reactions desorb from the catalytic electrode surface and diffuse into the electrolyte’s mainstream region, driven by the combined forces of convection and diffusion. Consequently, the mass transfer capacity of the fuel cell system critically determines the chemical reaction kinetics occurring at the catalytic electrode, thereby directly impacting the system’s overall performance. Presently, innovative strides have been made employing microfluidic technology to bolster the transfer dynamics of photons, electrons, and protons on a microscale, thus fostering the development of novel microfluidic PFCs [79,80]. These advancements effectively enhance pollutant removal efficacy and power generation efficiency. Nonetheless, it is worth noting that microfluidic PFC reactors and their operational control conditions possess inherent limitations when applied to large-scale wastewater treatment endeavors. To reduce the mass transport resistance, a PFC–membrane reactor system integrating PMS activation (PMS/PFC-MR) was reported by using the membrane as a cathode (Figure 3f,g). The results indicated that recycled filtration over the cathodic membrane accelerated the photo-electro catalysis process [64].

## 4. Other Applications of Photocatalytic Fuel Cells

**Figure 4:**
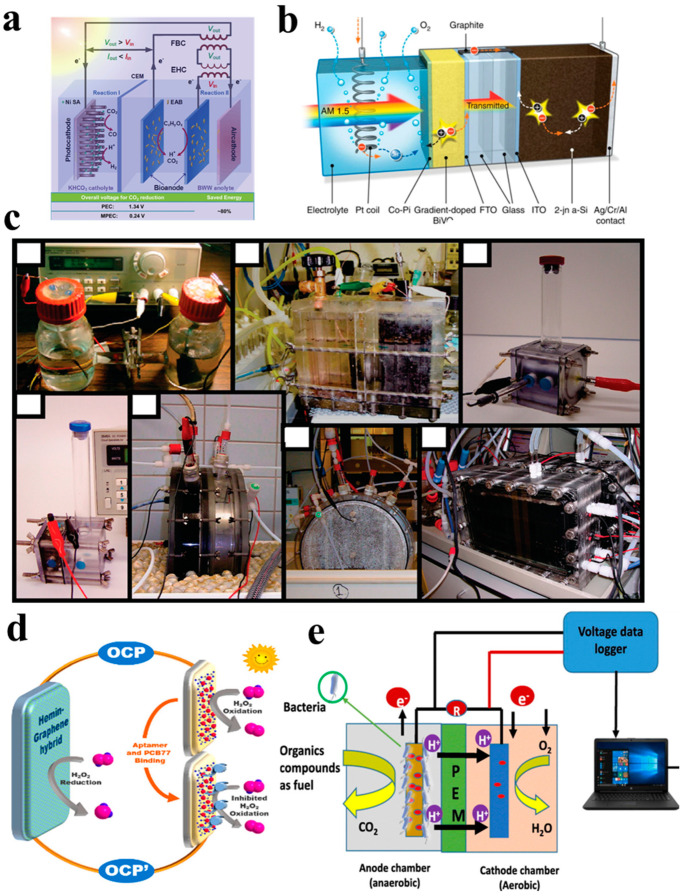
(**a**) Microbial photoelectrochemical system (MPEC) [81], (**b**) the combined device of solar cell [82], (**c**) MECs used in different studies [83], (**d**) proposed H_2_O_2_−based PFCs for self-powered [84], (**e**) MFCs−based biosensor [85].

In recent years, the development of PMEC systems has led to interdisciplinary advancements spanning environmental science, materials science, biology, and chemistry, among others. Consequently, their applications have diversified, with significant potential demonstrated in various fields such as wastewater treatment, hydrogen synthesis, carbon dioxide capture, environmental biosensors, wearable energy storage, and power generation devices [81,82,83,84,85] (Figure 4).

(1) Simultaneous CO_2_ reduction in wastewater treatment.

The research group led by Lovley [38] achieved the reduction of CO_2_ and the synthesis of various organic compounds using the cathodic biofilm *Sporomusa ovata* in MFC. This study demonstrates that the photovoltaic-driven microbial CO_2_ fixation method is more efficient in converting solar energy into organic products compared to traditional biomass techniques. Lu et al. [81] harnessed the action of anodic microorganisms to convert and recover the chemical energy contained in wastewater, supplying it to the photoanode to assist in the spontaneous reduction of CO_2_ to value-added fuels via cathodic photoelectrocatalysis. Li et al. [86] designed a dual-chamber PFC for CO_2_ self-driven self-circulation, where CO_2_ generated from wastewater degradation at the anode is spontaneously supplied to the rotating cathode under positive pressure and subsequently reduced to C1 value-added fuels via cathodic catalysis. Compared to a single photocatalytic system, the PFC system exhibited a 40% improvement in organic pollutant removal efficiency and a 6.7 μmol g^−1^ h^−1^ increase in the C1 fuel production rate. The formation of C_2_ products, such as ethylene (C_2_H_4_), and the generation of formic acid play pivotal roles in various chemical processes. Ethylene typically arises from reactions involving carbon atom rearrangements or carbon chain cleavages, while the generation of formic acid involves the bonding of carbon atoms with oxygen atoms. In-depth investigations into the formation mechanisms of these products contribute to a better understanding of the mechanism of PFCs/PMFCs.

(2) Simultaneous hydrogen production in wastewater treatment.

Hydrogen stands out as a promising clean energy source. Various studies have showcased PEC systems capable of hydrogen production through water splitting, albeit necessitating an applied bias voltage. Tang et al. [87] synthesized a photoelectrode utilizing a conductive carbon material through a hydrothermal loading process. Upon the irradiation of methyl orange (MO, 10 mg L^−1^) or berberine (BBR, 10 mg L^−1^) in a PFC environment setting for 2 h, the cell voltage stabilized at approximately 400 mV. Notably, the observed hydrogen production rate reached approximately 0.025 μmol cm^−2^, while the degradation and removal rates peaked at 98.2% and 90.1%, respectively. The application of bias at the two photoelectrodes effectively facilitated electron transfer from ZnFe_2_O_4_/Ag/Ag_3_VO_4_ to Fe-MoS_2_/rGO. This electron transfer process enabled the capture of protons by the electrons, ultimately leading to the generation of hydrogen. Moreover, the presence of pores and reactive oxygen species (ROS) facilitated the oxidation of organic matter, consequently mitigating photoinduced carrier recombination, and ultimately enhancing contaminant removal and hydrogen evolution efficiencies. Notably, Wang et al. [88] devised a self-biased PFC featuring a TiO_2_ photoanode, Nafion membrane, and platinum cathode. This innovative setup harnesses the plentiful electrons generated by the photocatalytic oxidation of formic acid to drive hydrogen production at the cathode, achieving an impressive Faraday efficiency of 88% [82,88,89,90]. Zhang et al. [91] developed a green supercapacitor-assisted PFC system, achieving a sustainable hydrogen generation under illumination (8 μmol g^−1^ h^−1^) and in the dark (3.25 μmol g^−1^ h^−1^).

(3) Environmental sensor.

Self-powered electrochemical sensors leverage suitable fuel cell setups to energize the detection process, capitalizing on the relationship between analyte concentration and fuel cell output performance to achieve sensing capabilities [85]. For example, Zhang et al. [84] constructed a visible-light-responsive self-powered sensing system utilizing a photo fuel cell for polychlorinated biphenyls (PCB77) detection. Here, nano gold-modified nitrogen-doped carbon acted as the photoanode, while a graphene–chlorine complex served as the cathode, with hydrogen peroxide serving as the substrate. The results revealed that the mercapto-functionalized adapters which assembled on the photoanode could selectively identify PCB77, displaying a robust linear correlation between its concentration and the output voltage.

(4) Wearable energy storage and power generation devices.

Wearable technology holds significant promise across a wide range of applications including smart devices, fitness tracking, smart home systems, and medical diagnostics. Harnessing the advantages of PFCs, an innovative flexible PFC. PFC [92] was constructed with an indium tin oxide (ITO) conductive film coated with P25 catalyst as the photoanode, while Pt/C serves as the cathode. It efficiently converts chemical energy from human metabolic processes and other waste substances in sweat into electricity, achieving a maximum power density of 4.0 mW cm^−2^g^−1^. This groundbreaking research showcases the application of PFCs in the realm of flexible wearable devices, addressing the limitations of enzyme biofuel cells. In a separate study, Qiu et al. [93] employed a combination of printing, electrochemical deposition, and screen printing techniques to develop a flexible planar integrated self-charging energy system, which consists of a PFC coupled with a micro-supercapacitor. Their findings demonstrate that the PFC, operating under light exposure and fueled by simulated wastewater, can continuously and efficiently charge the micro-supercapacitor while simultaneously degrading pollutants.

In order to generate a better understanding, various anode and cathode materials in the last five years have been listed, comparing their photocatalytic degradation, power generation performance, and mineralization rate with relevant reports, as shown in Table 1.

## 5. Conclusions

In this review, the fundamentals, working principle, and configuration development of PFCs and PMFCs were summarized. The most representative research papers specific to PFCs/PMFCs were highlighted in order to improve wastewater treatment in terms of coupled advanced oxidation technology, the rational design of high-efficiency electrodes, and the strengthening of the mass transfer process. Their applications in various fields such as hydrogen synthesis, carbon dioxide capture, environmental biosensors, wearable energy storage, and power generation devices were also reviewed. The development of new and efficient PFC/PMFC systems will improve the efficiency of wastewater co-removal and reduce energy consumption, which has an important scientific and application value for the treatment and resource recovery of refractory organic pollutants in wastewater.

## Figures and Tables

**Table 1:** PFC performance comparison.

Reaction Type	Photoanode	Pollutant	Concentration (%)	Degradation Time (min)	Pmax (mW/cm^2^)	Total Organic Carbon Removal Efficiency	Ref.
PFC + PMS	TiO_2_	Norfloxacin	98.1	60	0.019	72.3	[94]
	CuMnO	Rifampin	96.9	15	0.78	78.12	[95]
	MoS_2_/TiO_2_	Rhodamine B	69.16	300	0.114	/	[96]
	BiVO_4_	Methyl orange	93.78	120	0.0065	85.4	[97]
PFC	WO_3_/W	Methyl blue	91.6	90	0.34	/	[68]
	TiO_2_	Toluene	74%	120	0.02	/	[20]
	TNA-Cu	2-Methy l-4-chlorophenoxyacetic	93.7	80	0.0157		[19]
	TiO_2_	Methyl orange	92	60	0.074	/	[51]
	Fe@CdS/g-C_3_N_4_	Phenol	95.3	120	1.57	91	[98]
	g-C_3_N_4_/Fe^0^-WO_3_	Tetracycline	97.3	90	24	65.3	[99]
	ZnO/Bi_2_MoO_6_/MIL-101(Fe)	Tetracycline	95.1	90	9.25	89.6	[100]
	g-C_3_N_4_/TNAs	4-Chloronitrobenzene	97.7	300	5.52	/	[101]

## References

[1] Yu T., Yang B., Zhang R., Yang C., Yang C., Jiang J. (2024). Fabrication of a novel Z-S-scheme photocatalytic fuel cell with the Z-scheme TiO_2_/GO/g-C_3_N_4_ photoanode and S-scheme BiOAc1−xBr_x_/BiOBr photocathode for TC degradation. J. Mater. Sci. Technol..

[2] He Y., Chen K., Leung M., Zhang Y., Li L., Li G., Xuan J., Li J. (2022). Photocatalytic fuel cell—A review. Chem. Eng. J..

[3] Li M., Liu Y., Dong L., Shen C., Li F., Huang M., Ma C., Yang B., An X., Sand W. (2019). Recent advances on photocatalytic fuel cell for environmental applications—The marriage of photocatalysis and fuel cells. Sci. Total Environ..

[4] Tong Y., Wei J., Mo R., Ma H., Ai F. (2022). Photocatalytic Microbial Fuel Cells and Performance Applications: A Review. Front. Chem..

[5] Jordan M., Ojeda A., Larson E., Rogers S. (2023). Investigating the Relationship between Surface Water Pollution and Onsite Wastewater Treatment Systems. Environ. Sci. Technol..

[6] Karthik V., Senthil P., Vo D., Selvakumar P., Gokulakrishnan M., Keerthana P., Audilakshmi V., Jeyanthi J. (2021). Enzyme-loaded nanoparticles for the degradation of wastewater contaminants: A review. Environ. Chem. Lett..

[7] Yateh M., Li F., Tang Y., Li C., Xu B. (2024). Energy consumption and carbon emissions management in drinking water treatment plants: A systematic review. J. Clean. Prod..

[8] Kraaijeveld E., Rijsdijk S., van der Poel S., van der Hoek J., Rabaey K., van Halem D. (2024). Electrochemical arsenite oxidation for drinking water treatment: Mechanisms, by-product formation and energy consumption. Water Res..

[9] Mishra P., Saravanan P., Packirisamy G., Jang M., Wang C. (2021). A subtle review on the challenges of photocatalytic fuel cell for sustainable power production. Int. J. Hydrogen Energy.

[10] He Y., Yuan R., Leung M. (2019). Highly efficient AgBr/BiVO_4_ photoanode for photocatalytic fuel cell. Mater. Lett..

[11] Yan P., Shi H., Chen Y., Gao X., Fang F., Guo J. (2020). Optimization of recovery and utilization pathway of chemical energy from wastewater pollutants by a net-zero energy wastewater treatment model. Renew. Sust. Energ. Rev..

[12] Kant Bhatia S., Ahuja V., Chandel N., Mehariya S., Kumar P., Vinayak V., Saratale G., Raj T., Kim S., Yang Y. (2022). An overview on microalgal-bacterial granular consortia for resource recovery and wastewater treatment. Bioresour. Technol..

[13] Lee S., Ho L., Ong A., Wong Y., Voon C., Khalik W., Yusoff N., Nordin N. (2018). Exploring the relationship between molecular structure of dyes and light sources for photodegradation and electricity generation in photocatalytic fuel cell. Chemosphere.

[14] Vasseghian Y., Khataee A., Dragoi E., Moradi M., Nabavifard S., Oliveri Conti G., Mousavi Khaneghah A. (2020). Pollutants degradation and power generation by photocatalytic fuel cells: A comprehensive review. Arab. J. Chem..

[15] Mazumder J., Yoshikawa H., Miyake H., Shibata T., Tamiya E. (2017). Photocatalytic alginate fuel cells for energy production and refining of macroalgae. RSC Adv..

[16] Dhawle R., Mantzavinos D., Lianos P. (2021). UV/H_2_O_2_ degradation of diclofenac in a photocatalytic fuel cell. Appl. Catal. B Environ..

[17] Zhang J., Yang P., Zheng J., Li J., Lv S., Jin T., Zou Y., Xu P., Cheng C., Zhang Y. (2020). Degradation of gaseous HCHO in a rotating photocatalytic fuel cell system with an absorption efficiency of up to 94%. Chem. Eng. J..

[18] An X., Stelter D., Keyes T., Reinhard B. (2019). Plasmonic Photocatalysis of Urea Oxidation and Visible-Light Fuel Cells. Chem.

[19] Ye Y., Bruning H., Li X., Yntema D., Rijnaarts H. (2018). Significant enhancement of micropollutant photocatalytic degradation using a TiO_2_ nanotube array photoanode based photocatalytic fuel cell. Chem. Eng. J..

[20] Wang C., Liu Y., Chen R., Zhu X., Ye D., Yang Y., Liao Q. (2023). Gas diffusion TiO_2_ photoanode for photocatalytic fuel cell towards simultaneous VOCs degradation and electricity generation. J. Hazard. Mater..

[21] Ni J., Wen Y., Pan D., Bai J., Zhou B., Zhao S., Wang Z., Liu Y., Zeng Q. (2023). Light-driven simultaneous water purification and green energy production by photocatalytic fuel cell: A comprehensive review on current status, challenges, and perspectives. Chem. Eng. J..

[22] Miklos D., Remy C., Jekel M., Linden K., Drewes J., Hübner U. (2018). Evaluation of advanced oxidation processes for water and wastewater treatment—A critical review. Water Res..

[23] Kaneko M., Nemoto J., Ueno H., Gokan N., Ohnuki K., Horikawa M., Saito R., Shibata T. (2006). Photoelectrochemical reaction of biomass and bio-related compounds with nanoporous TiO_2_ film photoanode and O_2_-reducing cathode. Electrochem. Commun..

[24] Lui G., Jiang G., Fowler M., Yu A., Chen Z. (2019). A high performance wastewater-fed flow-photocatalytic fuel cell. J. Power Sources.

[25] Gu D., Zhang G., Zou J. (2021). High temperature thermo-photocatalysis driven carbon removal in direct biogas fueled solid oxide fuel cells. Chin. Chem. Lett..

[26] Jiang P., Zhou T., Bai J., Zhang Y., Li J., Zhou C., Zhou B. (2023). Nitrogen-containing wastewater fuel cells for total nitrogen removal and energy recovery based on Cl•/ClO• oxidation of ammonia nitrogen. Water Res..

[27] Xu Q., Qian B., Zhang Y., Li H., Wu Y. (2022). Heterostructure Fe_2_O_3_/BiVO_4_ as a Photoanode for a Visible-Light-Responsive Photocatalytic Fuel Cell. ACS Appl. Energy Mater..

[28] Xie S., Ouyang K., Shao Y. (2017). A solar responsive photocatalytic fuel cell with a heterostructured ZnFe_2_O_4_/TiO_2_-NTs photoanode and an air-breathing cathode. Int. J. Hydrogen Energy.

[29] Queiroz B., Fernandes J., Martins C., Wender H. (2022). Photocatalytic fuel cells: From batch to microfluidics. J. Environ. Chem. Eng..

[30] Tempelaere M., Zimmermann M., Chatenet M. (2023). 3D-structured electrocatalysts for improved mass-transfer in proton-exchange membrane fuel cell cathodes. Curr. Opin. Electrochem..

[31] Logan B., Rabaey K. (2012). Conversion of Wastes into Bioelectricity and Chemicals by Using Microbial Electrochemical Technologies. Science.

[32] Gupta S., Patro A., Mittal Y., Dwivedi S., Saket P., Panja R., Saeed T., Martínez F., Yadav A. (2023). The race between classical microbial fuel cells, sediment-microbial fuel cells, plant-microbial fuel cells, and constructed wetlands-microbial fuel cells: Applications and technology readiness level. Sci. Total Environ..

[33] Greenman J., Gajda I., Ieropoulos I. (2019). Microbial fuel cells (MFC) and microalgae; photo microbial fuel cell (PMFC) as complete recycling machines. Sustain. Energy Fuels.

[34] Bazina N., Ahmed T., Almdaaf M., Jibia S., Sarker M. (2023). Power generation from wastewater using microbial fuel cells: A review. J. Biotechnol..

[35] Lee C., Ha H., Ahn Y., Liu H. (2023). Performance of single-layer paper-based co-laminar flow microbial fuel cells. J. Power Sources.

[36] Kižys K., Zinovičius A., Jakštys B., Bružaitė I., Balčiūnas E., Petrulevičienė M., Ramanavičius A., Morkvėnaitė-Vilkončienė I. (2023). Microbial Biofuel Cells: Fundamental Principles, Development and Recent Obstacles. Biosensors.

[37] Leicester D., Settle S., McCann C., Heidrich E. (2023). Investigating Variability in Microbial Fuel Cells. Appl. Environ. Microbiol..

[38] Lu L., Williams N., Turner J., Maness P., Gu J., Ren Z. (2017). Microbial Photoelectrosynthesis for Self-Sustaining Hydrogen Generation. Environ. Sci. Technol..

[39] Hu X., Liu J., Cheng W., Li X., Zhao Y., Wang F., Geng Z., Wang Q., Dong Y. (2023). Synergistic interactions of microbial fuel cell and microbially induced carbonate precipitation technology with molasses as the substrate. Environ. Res..

[40] Khan M., Khan M., Min B., Cho M. (2018). Microbial fuel cell assisted band gap narrowed TiO_2_ for visible light-induced photocatalytic activities and power generation. Sci. Rep..

[41] Chen X., Shen S., Guo L., Mao S. (2010). Semiconductor-based Photocatalytic Hydrogen Generation. Chem. Rev..

[42] Li B., He Y., Xiao M., Zhang Y., Wang Z., Qin Z., Chai B., Yan J., Li J., Li J. (2022). A solar-light driven photocatalytic fuel cell for efficient electricity generation and organic wastewater degradation. Colloids Surf. A.

[43] Caglar A., Aktas N., Kivrak H. (2022). Photocatalytic glucose electrooxidation of titanium dioxide doped CdTe enhanced for a photocatalytic fuel cell. Fuel.

[44] Hu X., Qin J., Wang Y., Wang J., Yang A., Tsang Y.F., Liu B. (2022). Synergic degradation Chloramphenicol in photo-electrocatalytic microbial fuel cell over Ni/MXene photocathode. J. Colloid Interface Sci..

[45] Yu H., Xue Y., Liang S., Wang X. (2022). Preparation of a Z-system photocatalyst (oxygen-doped carbon nitride/nitrogen-doped carbon dots/bismuth tetroxide) and its application in a photocatalytic fuel cell. J. Photochem. Photobiol. A.

[46] John S., Nogala W., Gupta B., Singh S. (2022). Synergy of photocatalysis and fuel cells: A chronological review on efficient designs, potential materials and emerging applications. Front. Chem..

[47] Rej S., Hejazi S.M.H., Badura Z., Zoppellaro G., Kalytchuk S., Kment Š., Fornasiero P., Naldoni A. (2022). Light-Induced Defect Formation and Pt Single Atoms Synergistically Boost Photocatalytic H_2_ Production in 2D TiO_2_-Bronze Nanosheets. ACS Sustain. Chem. Eng..

[48] Shetti R., Sreenivasulu M., Mathi S., Shetti N.P. (2023). Highly Efficient and Low-Cost CuFeCN as an OER and HER Electrocatalyst for Sustainable Hydrogen Production. Energy Fuels.

[49] Ding Y., Li J., Yan K., Zhang J. (2022). A miniature self-powered electrochemical sensor for the determination of patulin based on an integrated photocatalytic fuel cell. Sens. Actuators B Chem..

[50] Yong Z., Lam S., Sin J., Zeng H., Mohamed A., Jaffari Z. (2022). Boosting sunlight-powered photocatalytic fuel cell with S-scheme Bi_2_WO_6_/ZnO nanorod array composite photoanode. Inorg. Chem. Commun..

[51] Liu Y., Chen R., Zhu X., Ye D., Yang Y., Li J., Wang D., An L., Liao Q. (2022). 3D radially-grown TiO_2_ nanotubes/Ti mesh photoanode for photocatalytic fuel cells towards simultaneous wastewater treatment and electricity generation. J. Clean. Prod..

[52] Pan D., Xiao S., Chen X., Li R., Cao Y., Zhang D., Pu S., Li Z., Li G., Li H. (2019). Efficient Photocatalytic Fuel Cell via Simultaneous Visible-Photoelectrocatalytic Degradation and Electricity Generation on a Porous Coral-like WO_3_/W Photoelectrode. Environ. Sci. Technol..

[53] Xie S., Ouyang K. (2017). Degradation of refractory organic compounds by photocatalytic fuel cell with solar responsive WO_3_/FTO photoanode and air-breathing cathode. J. Colloid Interface Sci..

[54] Wang B., Zhang H., Lu X., Xuan J., Leung M. (2014). Solar photocatalytic fuel cell using CdS–TiO_2_ photoanode and air-breathing cathode for wastewater treatment and simultaneous electricity production. Chem. Eng. J..

[55] Zhang J., Li L., Zheng J., Yang P., Wu X., Cheng C., Li J., Tian Y., Wang F. (2019). Improved organic pollutants removal and simultaneous electricity production via integrating Fenton process and dual rotating disk photocatalytic fuel cell system using bamboo charcoal cathode. Chem. Eng. J..

[56] Li K., Zhang H., Tang T., Tang Y., Wang Y., Jia J. (2016). Facile electrochemical polymerization of polypyrrole film applied as cathode material in dual rotating disk photo fuel cell. J. Power Sources.

[57] Xu P., Zheng D., Xu H. (2019). The feasibility and mechanism of reverse electrodialysis enhanced photocatalytic fuel cell-Fenton system on advanced treatment of coal gasification wastewater. Sep. Purif. Technol..

[58] Chen R., Xia M., Zhu X., Liao Q., Ye D., An L., Yu Y., Jiao L., Zhang W. (2018). A visible-light responsive micro photocatalytic fuel cell with laterally arranged electrodes. Appl. Therm. Eng..

[59] Liu J., Xia M., Chen R., Zhu X., Liao Q., Ye D., Zhang B., Zhang W., Yu Y. (2019). A membrane-less visible-light responsive micro photocatalytic fuel cell with the laterally-arranged CdS/ZnS-TiO_2_ photoanode and air-breathing CuO photocathode for simultaneous wastewater treatment and electricity generation. Sep. Purif. Technol..

[60] Sun Q., Wu S., You D., Zang T., Dong L. (2019). Novel composite functional photocatalytic fuel cell assisted by Fenton-like reactions. Appl. Surf. Sci..

[61] Li J., Li J., Chen Q., Bai J., Zhou B. (2013). Converting hazardous organics into clean energy using a solar responsive dual photoelectrode photocatalytic fuel cell. J. Hazard. Mater..

[62] Oli H., Kim A., Park M., Bhattarai D., Pant B. (2022). Photocatalytic Fuel Cells for Simultaneous Wastewater Treatment and Power Generation: Mechanisms, Challenges, and Future Prospects. Energies.

[63] Zhang Y., Liu L., Chen Q., He Y., Leung M. (2019). Electricity generating & high efficiency advanced oxidation process including peroxymonosulfate activation in photocatalytic fuel cell. Chem. Eng. J..

[64] Zhang Y., Chen Q., Liu L., Wang Y., Leung M. (2020). Activation of peroxymonosulfate and recycled effluent filtration over cathode membrane CNFs-CoFe_2_O_4_/PVDF in a photocatalytic fuel cell for water pollution control. Chem. Eng. J..

[65] Feng C., Li F., Mai H., Li X. (2010). Bio-Electro-Fenton Process Driven by Microbial Fuel Cell for Wastewater Treatment. Environ. Sci. Technol..

[66] Zeng Q., Bai J., Li J., Li L., Xia L., Zhou B., Sun Y. (2018). Highly-stable and efficient photocatalytic fuel cell based on an epitaxial TiO_2_/WO_3_/W nanothorn photoanode and enhanced radical reactions for simultaneous electricity production and wastewater treatment. Appl. Energy.

[67] Zhao K., Zeng Q., Bai J., Li J., Xia L., Chen S., Zhou B. (2017). Enhanced organic pollutants degradation and electricity production simultaneously via strengthening the radicals reaction in a novel Fenton-photocatalytic fuel cell system. Water Res..

[68] Xu P., Xu H., Zheng D. (2019). Simultaneous electricity generation and wastewater treatment in a photocatalytic fuel cell integrating electro-Fenton process. J. Power Sources.

[69] Li J., Xu M., Yao G., Lai B. (2018). Enhancement of the degradation of atrazine through CoFe_2_O_4_ activated peroxymonosulfate (PMS) process: Kinetic, degradation intermediates, and toxicity evaluation. Chem. Eng. J..

[70] Lee S., Ho L., Ong S., Wong Y., Voon C., Khalik W., Yusoff N., Nordin N. (2017). A highly efficient immobilized ZnO/Zn photoanode for degradation of azo dye Reactive Green 19 in a photocatalytic fuel cell. Chemosphere.

[71] Li X., Chen S., Angelidaki I., Zhang Y. (2018). Bio-electro-Fenton processes for wastewater treatment: Advances and prospects. Chem. Eng. J..

[72] Chen X., Yao J., Dong H., Hong M., Gao N., Zhang Z., Jiang W. (2021). Enhanced bezafibrate degradation and power generation via the simultaneous PMS activation in visible light photocatalytic fuel cell. Water Res..

[73] Sun J., Liu L., Yang F. (2022). A visible-light-driven photocatalytic fuel cell/peroxymonosulfate (PFC/PMS) system using blue TiO_2_ nanotube arrays (TNA) anode and Cu-Co-WO_3_ cathode for enhanced oxidation of organic pollutant and ammonium nitrogen in real seawater. Appl. Catal. B Environ..

[74] Mikrut P., Mitoraj D., Beranek R., Macyk W. (2021). Facet-dependent activity of tailored anatase TiO_2_ crystals in photoanodes for photocatalytic fuel cells. Appl. Surf. Sci..

[75] Rastogi A., Al-Abed S., Dionysiou D. (2009). Sulfate radical-based ferrous–peroxymonosulfate oxidative system for PCBs degradation in aqueous and sediment systems. Appl. Catal. B Environ..

[76] Amor C., Fernandes J., Lucas M., Peres J. (2021). Hydroxyl and sulfate radical advanced oxidation processes: Application to an agro-industrial wastewater. Environ. Technol..

[77] Zhang M., Gong Y., Ma N., Zhao X. (2020). Promoted photoelectrocatalytic degradation of BPA with peroxymonosulfate on a MnFe_2_O_4_ modified carbon paper cathode. Chem. Eng. J..

[78] Modestino M., Hashemi S., Haussener S. (2016). Mass transport aspects of electrochemical solar-hydrogen generation. Energy Environ. Sci..

[79] Wang H., Chen X., Chen R., Zhu X., Liao Q., Ye D., Zhang B., Yu Y., Zhang W., Li J. (2019). A ternary hybrid CuS/Cu_2_O/Cu nanowired photocathode for photocatalytic fuel cell. J. Power Sources.

[80] Liu Z., Ye D., Chen R., Zhang B., Zhu X., Li J., Liao Q. (2018). A woven thread-based microfluidic fuel cell with graphite rod electrodes. Int. J. Hydrogen Energy.

[81] Lu L., Li Z., Chen X., Wang H., Dai S., Pan X., Ren Z.J., Gu J. (2020). Spontaneous Solar Syngas Production from CO_2_ Driven by Energetically Favorable Wastewater Microbial Anodes. Joule.

[82] Abdi F., Han L., Smets A., Zeman M., Dam B., van de Krol R. (2013). Efficient solar water splitting by enhanced charge separation in a bismuth vanadate-silicon tandem photoelectrode. Nat. Commun..

[83] Logan B., Call D., Cheng S., Hamelers H., Sleutels T., Jeremiasse A., Rozendal R. (2008). Microbial Electrolysis Cells for High Yield Hydrogen Gas Production from Organic Matter. Environ. Sci. Technol..

[84] Yan K., Zhu Y., Ji W., Chen F., Zhang J. (2018). Visible Light-Driven Membraneless Photocatalytic Fuel Cell toward Self-Powered Aptasensing of PCB77. Anal. Chem..

[85] Do M., Ngo H., Guo W., Chang S., Nguyen D., Liu Y., Varjani S., Kumar M. (2020). Microbial fuel cell-based biosensor for online monitoring wastewater quality: A critical review. Sci. Total Environ..

[86] Zhang J., Lv S., Zheng J., Yang P., Li J., Wu X., Jin T., Cheng C., Song Y., Li L. (2020). Self-CO_2_ Recycling Photocatalytic Fuel Cell for Enhancing Degradation of Pollutants and Production of Carbon-Neutral Fuel. ACS Sustain. Chem. Eng..

[87] Tang L., Liu L., Chen Q., Yang F., Quan X. (2020). The construction and performance of photocatalytic-fuel-cell with Fe-MoS2/reduced graphene oxide@carbon fiber cloth and ZnFe_2_O_4_/Ag/Ag_3_VO_4_@carbon felt as photo electrodes. Electrochim. Acta.

[88] Yuranov I., Autissier N., Sordakis K., Dalebrook A., Grasemann M., Orava V.P., Cendula P., Gubler L., Loren G. (2018). Heterogeneous Catalytic Reactor for Hydrogen Production from Formic Acid and Its Use in Polymer Electrolyte Fuel Cells. ACS Sustain. Chem. Eng. J..

[89] Shi X., Choi I., Zhang K., Kwon J., Kim D., Lee J., Oh S., Kim J., Park J. (2014). Efficient photoelectrochemical hydrogen production from bismuth vanadate-decorated tungsten trioxide helix nanostructures. Nat. Commun..

[90] Ye K., Li H., Huang D., Xiao S., Qiu W., Li M., Hu Y., Mai W., Ji H., Yang S. (2019). Enhancing photoelectrochemical water splitting by combining work function tuning and heterojunction engineering. Nat. Commun..

[91] Zhang J., Zheng J., Yang W. (2021). Green supercapacitor assisted photocatalytic fuel cell system for sustainable hydrogen production. Chem. Eng. J..

[92] Lui G., Jiang G., Lenos J., Lin E., Fowler M., Yu A., Chen Z. (2017). Advanced Biowaste-Based Flexible Photocatalytic Fuel Cell As a Green Wearable Power Generator. Adv. Mater. Technol..

[93] Qiu M., Sun P., Cui G., Tong Y., Mai W. (2019). A Flexible Microsupercapacitor with Integral Photocatalytic Fuel Cell for Self-Charging. ACS Nano.

[94] Li J., Li R., Zou L., Liu X. (2019). Efficient Degradation of Norfloxacin and Simultaneous Electricity Generation in a Persulfate-Photocatalytic Fuel Cell System. Catalysts.

[95] Sun Y., Xia L., Wang Y., Yao W., Wu Q., Min Y., Xu Q. (2023). Efficient rifampicin degradation and simultaneous energy recovery in photocatalytic fuel cell based on the enhanced PMS and H_2_O_2_ synergistic activation on sulfur-doped CuMnO/carbon felt cathode. Sep. Purif. Technol..

[96] Gu Z., Zhou J., An X., Chen Q., Hu C., Liu H., Qu J. (2021). A dual-biomimetic photocatalytic fuel cell for efficient electricity generation from degradation of refractory organic pollutants. Appl. Catal. B Environ..

[97] Shen L., Sun Y., Qin Q., Su Y., Wang L., Xia L., Lin S., Yao W., Wu Q., Min Y. (2022). The significant role of few sulfite replenishment in boosting electricity generation and organic wastewater degradation in photocatalytic fuel cell. Sep. Purif. Technol..

[98] Ammar S., Shafi R., Ali A. (2020). A novel airlift photocatalytic fuel cell (APFC) with immobilized CdS coated zerovalent iron (Fe@CdS) and g-C_3_N_4_ photocatalysts film as photoanode for power generation and organics degradation. Colloids Surf. A.

[99] Rabé K., Liu L., Nahyoon N.A. (2020). Electricity generation in fuel cell with light and without light and decomposition of tetracycline hydrochloride using g-C_3_N_4_/Fe^0^(1%)/TiO_2_ anode and WO_3_ cathode. Chemosphere.

[100] Hajiali M., Farhadian M., Tangestaninejad S. (2022). Enhance performance ZnO/Bi_2_MoO_6_/MIL-101(Fe) grown on fluorine-doped tin oxide as photoanode and CuO/Cu_2_O based on Cu mesh photocathode in the photocatalytic fuel cell. Energy Convers. Manag..

[101] Liu W., Huo S., Liu X., Wang Y., Xin S., Fu W., Gao M., Xie H. (2023). Coupling photocatalytic fuel cell based on S-scheme g-C_3_N_4_/TNAs photoanode with H_2_O_2_ activation for p-chloronitrobenzene degradation and simultaneous electricity generation under visible light. Sep. Purif. Technol..

